# Partial Cutting of Sternothyroid Muscle during Total Thyroidectomy: Impact on Postoperative Vocal Outcomes

**DOI:** 10.1155/2013/416535

**Published:** 2013-09-23

**Authors:** Hyoung Shin Lee, Sung Won Kim, Hyo Sang Park, Chan Woo Park, Ji Soo Kim, Jong Chul Hong, Yong-Ki Kim, Seon Mi Baek, Kang Dae Lee

**Affiliations:** ^1^Department of Otolaryngology-Head and Neck Surgery, Kosin University College of Medicine, 34 Amnam-Dong, Seo-Gu, Busan 602-702, Republic of Korea; ^2^Department of Otolaryngology-Head and Neck Surgery, Dong-A University College of Medicine, Busan 602-715, Republic of Korea; ^3^Kim Yong Ki Internal Medicine Clinic, Busan 602-808, Republic of Korea; ^4^Sharing and Happiness Hospital, Busan 612-035, Republic of Korea

## Abstract

*Background*. Cutting the sternothyroid (ST) muscle is a useful technique to expose the superior pole of thyroid gland during thyroidectomy. In this study, we evaluated the impact of partial cutting of the ST muscle on postoperative vocal outcomes after total thyroidectomy. *Methods*. A retrospective review of 57 patients who underwent total thyroidectomy with central neck dissection for micropapillary thyroid carcinoma was conducted. Group A (*n* = 26) included those without cutting the ST muscle, while group B (*n* = 31) included patients whose muscle was partially cut at the superior pole. All patients underwent voice analysis before the operation and 2 weeks and 1 month after the surgery, and the outcomes were compared between the two groups. *Results*. There were no differences between the two groups regarding the outcomes at each time of voice analysis. Group A showed a decrease of maximum frequency 2 weeks after surgery but showed no difference after 1 month. Group B showed a mild decrease in maximum frequency 2 weeks after surgery, but the difference was not significant. *Conclusion*. Partial cutting of ST muscle during thyroidectomy is useful to expose the superior pole without significant negative impact on postoperative outcomes of vocal analysis.

## 1. Introduction

Postoperative vocal outcomes after thyroidectomy may be one of the critical concerns for both the surgeon and the patient [1]. Temporary or permanent injury of the recurrent laryngeal nerve or the external branch of the superior laryngeal nerve may be the typical causes of vocal dysfunction after thyroidectomy. However, most of the voice alterations after thyroidectomy which has been reported in a variable incidence of 16~89% [2, 3] are considered to be not related to neural damage [2, 4]. The possible causes of voice change after thyroidectomy with preservation of the superior and recurrent laryngeal nerve have been described as division or injury of the strap muscles [2, 5, 6], cricothyroid muscle injury [2], modified vascular supply and venous drainage of the larynx [6], and mucosal congestion due to orotracheal intubation [4]. Local pain in the neck and the psychological reaction to the postoperative situation have been considered as other causes of postthyroidectomy voice change [6]. These factors imply that preventing damage to extralaryngeal muscular structures may be related to decrease of postoperative voice change.

Partial cutting of sternothyroid (ST) muscle during thyroidectomy is a well-established surgical technique to expose the superior pole of the thyroid gland, especially in patients with large thyroid gland [2, 7, 8]. The technique has been considered to facilitate isolation and ligation of the vascular pedicles of superior pole and allow better identification and preservation of the external branch of the superior laryngeal nerve [7]. Some surgeons even conduct it as a routine surgical procedure during thyroidectomy [8, 9]. While a contributory role of strap muscles in voice production has been suggested [7, 10], impact of the procedure on postthyroidectomy voice outcome is controversial [7, 8, 10]. In this study, we evaluated the impact of partial cutting of ST muscle on postoperative outcomes of vocal analysis after total thyroidectomy.

## 2. Materials and Methods

### 2.1. Patients

A retrospective review of 57 patients who underwent total thyroidectomy with central neck dissection for micropapillary thyroid carcinoma between October 2012 and January 2013 was considered eligible for this study. All the patients underwent acoustic voice and aerodynamic analysis before the operation and 2 weeks and 1 month after the surgery. Exclusion criteria included patients with preoperative vocal cord paralysis, benign lesions of vocal cord, speech disorders, history of previous neck surgery, or irradiation. Patients with postoperative vocal cord paralysis or evidence of external branch of superior laryngeal nerve injury were also excluded from the study. Social history of alcohol drinking and smoking was acquired. Drinking alcohol or smoking at least once a week was considered as inclusion criteria. Patients with reflux symptom index greater than 13 were considered to have laryngopharyngeal reflux disease (LPRD). This study was approved by the institutional review board of our center (KUGH IRB 12-138).

### 2.2. Surgical Technique of Thyroidectomy

**Figure 1:**
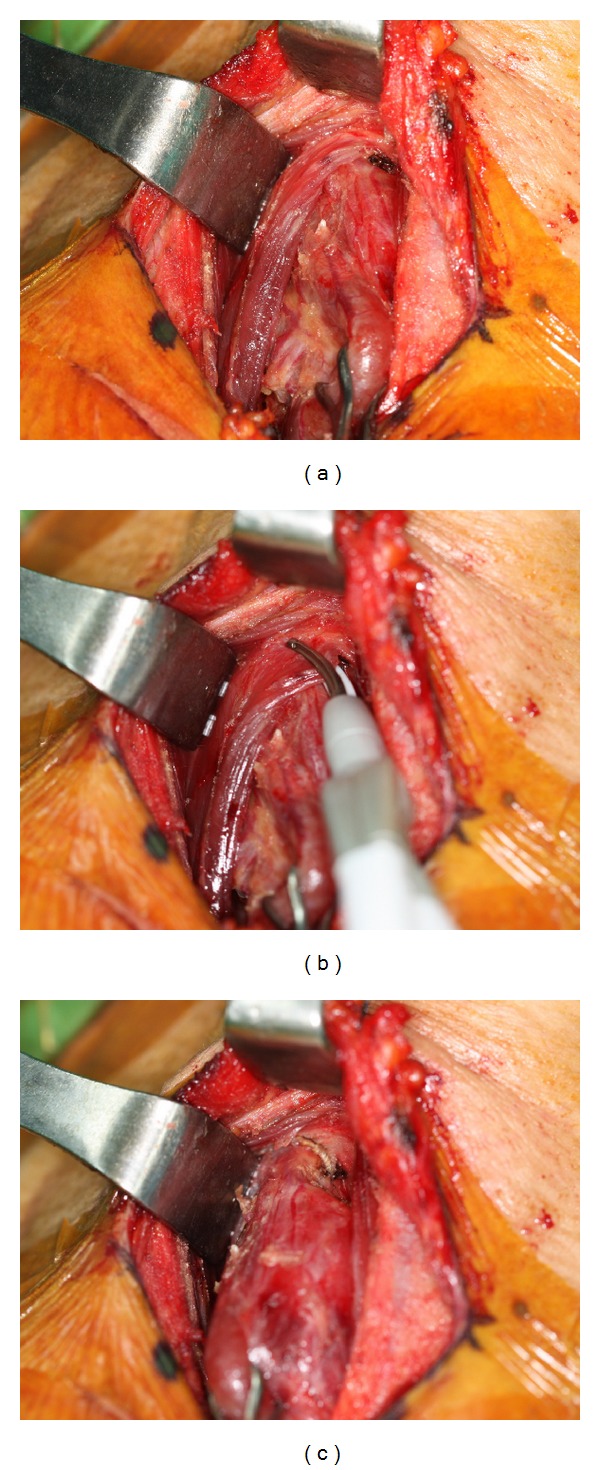
Partial cutting of sternothyroid muscle during thyroidectomy. (a) Superior pole of right thyroid gland covered by sternothyroid muscle. (b) Cutting the medical 2 cm of sternothyroid muscle. (c) Good exposure of the superior pole.

Thyroidectomy was conducted according to the standardized conventional technique. The strap muscles were divided along the linea alba and separated from the thyroid gland. Lateral aspect of the thyroid was exposed by ligation of the middle thyroid vein and lateral retraction of the strap muscles. The recurrent laryngeal nerve was identified in all cases at the lower pole of thyroid, and dissection of the gland was conducted along the nerve from caudal to cranial direction. Superior pole of the gland was exposed by pulling the thyroid gland toward inferior and medial direction and retracting the strap muscles laterally. At this point, the surgeon decided whether to cut the ST muscle or not. If surgical exposure of the upper end of the superior pole was insufficient, medial edge of ST muscle (approximately 2 cm) was cut using Harmonic Curved Shears (Harmonic Focus; Johnson & Johnson Medical, Cincinnati, OH, USA) at the laryngeal head where the muscle inserts along the thyroid cartilage (Figure 1). Reapproximation of the divided muscle was not conducted. After removing the bilateral thyroid gland, the strap muscles were reapproximated at the midline, and meticulous skin closure was conducted.

### 2.3. Acoustic and Aerodynamic Analysis

Acoustic analysis was performed by a speech pathologist using Computerized Speech Lab (CSL, KAY Electrics Corp, Model 4500, NJ, USA). Voice range profile (VRP) was examined from phonation from the lowest to the highest note after adequate inspiration. The software screen presented the notes on a piano keyboard demonstrating the minimum frequency (*F*_min⁡_), maximum frequency (*F*_max⁡_), and the range of frequency (*F*_range_). The minimum intensity (Min-dB), maximum intensity (Max-dB), and the range of intensity (dB-range) were also assessed during the phonation. Using the multidimensional voice program (MDVP), the fundamental frequency (*F*_0_), jitter, shimmer, and noise-to-harmonic ratio (NHR) were evaluated. The patient was seated on a chair and told to say “a” for 3 seconds at a comfortable level of effort, and the voice was recorded with a microphone 10 cm away from the lips. Maximal phonation time (MPT) was assessed during phonation of “a” at a constant pitch and intensity after full inspiration. The maximum value among three consecutive examinations of MPT was taken. 

### 2.4. Perceptual Analysis

Perceptual analysis was conducted by a single expert speech therapist of our center using a modified four scale of the GRBAS scale proposed by Hirano [11]. The scale varied from 0, normal, to 3, severe. In this study, only the parameter G (grade) from the scale was included to quantify the voice quality. 

### 2.5. Statistical Analysis

All statistical analyses were conducted with PASW 18 software (SPSS Inc., Chicago, IL, USA). Independent samples *t*-test was performed to compare the numeric data between two groups, and paired samples *t*-test was conducted to analyze the postoperative change of the parameters in each group. Fisher's exact test was used for analysis of categorical data. A two-sided *P* value <0.05 was considered to be statistically significant. 

## 3. Results

There were no significant differences between the two groups regarding age, sex, smoking and drinking habits, and LPRD. The outcomes of acoustic analysis showed no difference between the groups at each time of assessment (Table 1). 

**Figure 2:**
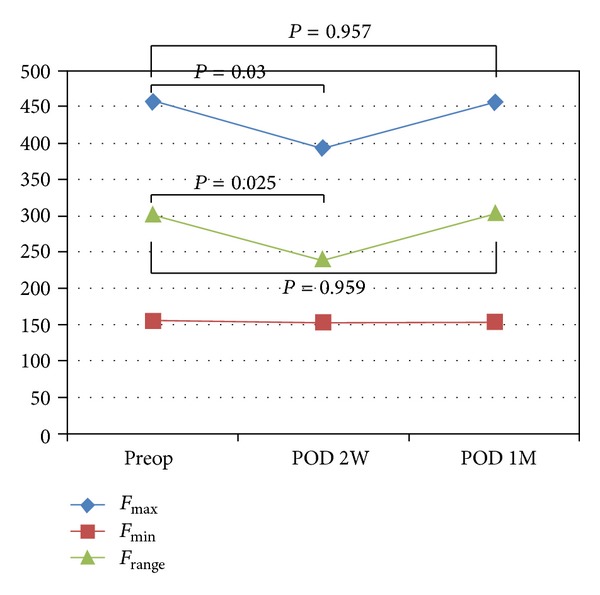
Mean value of frequency in group A. *F*_max⁡_, maximum frequency; *F*_min⁡_, minimum frequency; *F*_range_, range of frequency; Preop, preoperative; POD, postoperative day; 2W, 2 weeks; and 1M, 1 month.

**Figure 3:**
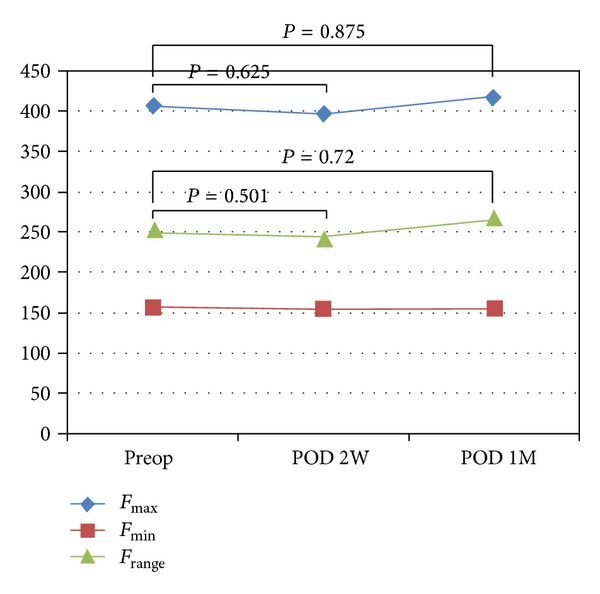
Mean value of frequency in group B. *F*_max⁡_, maximum frequency; *F*_min⁡_, minimum frequency; *F*_range_, range of frequency; Preop, preoperative; POD, postoperative day; 2W, 2 weeks; and 1M, 1 month.

Vocal outcomes of group A (Table 2) showed decreased *F*_max⁡_ and *F*_range_ at 2 weeks after surgery. However, the value of both parameters increased 1 month after surgery showing no difference compared to those assessed preoperatively (Figure 2). Other parameters were unremarkable. On the other hand, group B demonstrated no significant change after the surgery regarding the assessed parameters (Table 3). *F*_max⁡_ showed a mild decrease 2 weeks after the surgery but with no statistical significance (Figure 3). 

## 4. Discussion

Role of ST muscle on vocal fold is controversial. Sonninen [12] presented that ST muscle contributed to the lengthening of the vocal folds by ventrodorsal gliding in the cricothyroid articulation resulting in forward movement of the thyroid cartilage. On the contrary, Schilling [13] considered it as a main shortener of the vocal folds, acting as an antagonist to the cricothyroid muscle. The mechanism to shorten the vocal folds has been demonstrated by the greater torque of ST muscle contraction on the posterior side of the rotation axis of the cricothyroid articulation than on the anterior side. The attachment of ST muscle to the linea obliqua located at the posterior aspect of the thyroid cartilage may explain such a hypothesis. Despite the controversy on the impact of ST muscle on the vocal fold, correlation between the division of the ST muscle and postoperative vocal outcome has been assumed [14].

Sonninen [10] in 1956, demonstrated that bilateral division of strap muscles during thyroidectomy resulted in decreased pitch which could not be recovered. On the other hand, Jaffe and Young [7] showed that strap muscle division does not result in any subjective or objective functional sequelae. The functional impact of cutting ST muscle during thyroidectomy on postoperative voice was reported by Henry et al. [8] in 2008. They showed no significant difference in both voice symptoms and aerodynamic parameters. However, the patient group was enrolled from two different institutes with opposing preferences for cutting the ST muscle. Moreover, the extent of thyroidectomy whether it was lobectomy or total thyroidectomy and the extent of division of strap muscles whether it was partial or complete were not described. We assume that this may have led to a selection bias. Nonetheless, the vocal outcomes regarding division of ST muscle coincide with our study.  Mildly decreased fundamental frequency just after surgery and recovery 3 months later matches up with our result of recovery 1 month after surgery. In fact, our study revealed that the vocal outcomes after partial cutting of ST muscle during total thyroidectomy had no difference in postoperative progress compared to the studies [1, 2, 5, 6] on voice outcomes after thyroidectomy without neural injury. 

Voice outcome after thyroidectomy in patients with preservation of the recurrent and superior laryngeal nerve with normal vocal fold mobility has been reported by several authors. Debruyne et al. [6] showed a tendency of lower speaking fundamental frequency, smaller pitch range, increased jitter, and a decreased harmonic prominence on postoperative day 4, which all disappeared on day 15 except 2 patients with lower speaking fundamental frequency. Sinagra et al. [2] reported decreased mean fundamental frequency after thyroidectomy which recovered in the fourth and sixth postoperative months. Lombardi et al. [1] demonstrated the subjective impairment of voice after thyroidectomy which was evaluated with a questionnaire. Patients showed subjective impairment of voice up to 1 month after surgery but showed improvement 3 months after surgery. However, the objective parameters obtained from the MDVP and VRP showed no difference in postoperative outcomes. 

The limitation of this study may be the limited numbers of patients and the absence of evaluation for subjective symptoms of the patients. Roubeau et al. [15] insisted that strap muscles are involved in the evolution of the rise and fall of vocal frequency rather than the pitch itself. Therefore, evaluation of postoperative singing voice may be required to justify the safety of partial cutting of ST muscle during thyroidectomy [16]. In addition, preservation of the recurrent laryngeal nerve was confirmed with the laryngoscope in this study, but the preservation of the external branch of the superior laryngeal nerve should be validated by conducting electromyogram (EMG) of the CT muscle. In fact, we are conducting a prospective study to evaluate the impact of partial cutting of the ST muscle on postoperative vocal outcomes (subjective and objective parameters) after thyroidectomy, which excludes patients with denervation signal on postoperative EMG of the CT muscle.

In conclusion, partial cutting of ST muscle during total thyroidectomy may be applied to facilitate the exposure and dissection of the superior pole without concerns for significant deterioration of postoperative phonation. However, additional prospective studies considering more clinical variables and more parameters to evaluate postthyroidectomy vocal outcomes may be required to validate the safety of the procedure.

## Figures and Tables

**Table 1:** Comparison of patient factors and acoustic and perceptual analyses of groups A and B.

Parameter	Group A (*n* = 26)	Group B (*n* = 31)	*P*
Age (years)	50.88 ± 7.157	50.97 ± 9.383	0.971
Sex (male : female)	6 : 20	4 : 27	0.486
Smoking (%)	3 (12.0%)	1 (3.7%)	0.341
Alcohol (%)	4 (16.0%)	1 (3.7%)	0.183
LPRD (%)	9 (37.5%)	6 (22.2%)	0.232
Preop-*F*_0_ (Hz)	196.26 ± 42.71	191.38 ± 39.61	0.657
POD 2W-*F*_0_ (Hz)	195.67 ± 49.16	186.91 ± 35.49	0.439
POD 1M-*F*_0_ (Hz)	193.79 ± 47.59	185.55 ± 45.44	0.511
Preop-jitter (%)	0.57 ± 0.37	0.85 ± 1.56	0.369
POD 2W-jitter (%)	0.73 ± 0.54	0.90 ± 1.73	0.639
POD 1M-jitter (%)	1.52 ± 4.19	0.52 ± .0.27	0.198
Preop-shimmer (%)	3.54 ± 1.78	3.10 ± 1.61	0.330
POD 2W-shimmer (%)	3.45 ± 1.59	3.26 ± 1.05	0.594
POD 1M-shimmer (%)	2.88 ± 1.66	2.91 ± 1.17	0.930
Preop-NHR (dB)	0.14 ± 0.02	0.12 ± 0.02	0.919
POD 2W-NHR (dB)	0.16 ± 0.15	0.13 ± 0.01	0.311
POD 1M-NHR (dB)	0.13 ± 0.02	0.12 ± 0.01	0.671
Preop-MPT (sec)	14.75 ± 5.50	15.40 ± 4.93	0.636
POD 2W-MPT (sec)	14.48 ± 5.30	13.79 ± 4.27	0.592
POD 1M-MPT (sec)	15.89 ± 6.46	14.57 ± 6.56	0.454
Preop-GRBAS grade	0.35 ± 0.56	0.42 ± 0.45	0.587
POD 2W-GRBAS grade	0.60 ± 0.66	0.45 ± 0.44	0.346
POD 1M-GRBAS grade	0.42 ± 0.66	0.34 ± 0.37	0.566

LPRD: laryngopharyngeal reflux disease; Preop: preoperative; POD: postoperative day; *F*_0_: fundamental frequency; 2W: 2 weeks; 1M: 1 month; NHR: noise-to-harmonic ratio; and MPT: maximal phonation time.

**Table 2:** Comparison of preoperative and postoperative outcomes of various vocal analysis in group A.

Analysis	Preop	POD 2 weeks	*P*	POD 1 month	*P*
*F*_0_ (Hz)	196.26 ± 42.71	195.67 ± 49.16	0.901	193.79 ± 47.59	0.483
Jitter (%)	0.57 ± 0.37	0.73 ± 0.54	0.208	1.52 ± 4.20	0.263
Shimmer (%)	3.54 ± 1.78	3.45 ± 1.59	0.804	2.88 ± 1.66	0.115
NHR (dB)	0.12 ± 0.02	0.16 ± 0.15	0.251	0.13 ± 0.18	0.627
MPT (sec)	14.75 ± 5.50	14.48 ± 5.30	0.789	15.89 ± 6.46	0.368
*F*_max⁡_ (Hz)	457.97 ± 157.07	392.77 ± 131.16	**0.030**	456.63 ± 190.72	0.957
*F*_min⁡_ (Hz)	156.31 ± 35.35	152.79 ± 39.85	0.430	153.78 ± 38.15	0.392
*F*_range_ (Hz)	301.54 ± 142.86	239.98 ± 101.50	**0.025**	302.85 ± 177.31	0.959
Max-dB	90.85 ± 5.66	89.81 ± 6.51	0.382	91.08 ± 6.27	0.805
Min-dB	69.96 ± 7.64	71.00 ± 5.38	0.487	71.42 ± 5.89	0.390
dB-range	20.88 ± 7.47	18.69 ± 5.985	0.101	19.65 ± 8.79	0.404
GRBAS grade	0.35 ± 0.56	0.60 ± 0.66	0.085	0.42 ± 0.66	0.557

Preop: preoperative; POD: postoperative day; *F*_0_: fundamental frequency; NHR: noise-to-harmonic ratio; MPT: maximum phonation time; *F*_max⁡_: maximum frequency, *F*_min⁡_: minimum frequency; *F*_range_: range of frequency; Max-dB: maximum intensity; Min-dB: minimum intensity; and dB-range: range of intensity.

**Table 3:** Comparison of preoperative and postoperative outcomes of various vocal analysis in group B.

Analysis	Preop	POD 2 weeks	*P*	POD 1 months	*P*
*F*_0_ (Hz)	191.38 ± 39.61	186.91 ± 35.49	0.372	185.55 ± 45.44	0.291
Jitter (%)	0.85 ± 1.56	0.91 ± 1.75	0.895	0.52 ± 0.27	0.238
Shimmer (%)	3.10 ± 1.61	3.26 ± 1.05	0.572	2.92 ± 1.17	0.435
NHR (dB)	0.12 ± 0.17	0.13 ± 0.01	0.085	0.12 ± 0.01	0.554
MPT (sec)	15.52 ± 4.97	13.79 ± 4.27	0.068	14.57 ± 6.56	0.411
*F*_max⁡_ (Hz)	407.29 ± 146.82	395.78 ± 146.27	0.625	416.92 ± 139.49	0.875
*F*_min⁡_ (Hz)	155.69 ± 27.81	152.85 ± 31.16	0.501	153.38 ± 33.62	0.720
*F*_range_ (Hz)	251.61 ± 130.69	242.93 ± 128.00	0.675	267.43 ± 136.73	0.135
Max-dB	88.81 ± 7.34	88.52 ± 5.64	0.821	90.91 ± 4.89	0.077
Min-dB	71.26 ± 4.86	72.26 ± 5.75	0.477	68.60 ± 6.42	0.106
dB-range	17.55 ± 7.07	16.26 ± 4.82	0.262	20.70 ± 6.31	0.150
GRBAS grade	0.42 ± 0.45	0.45 ± 0.44	0.787	0.34 ± 0.37	0.134

Preop: preoperative; POD: postoperative day; *F*_0_: fundamental frequency; NHR: noise-to-harmonic ratio; MPT: maximum phonation time; *F*_max⁡_: maximum frequency, *F*_min⁡_: minimum frequency; *F*_range_: range of frequency; Max-dB: maximum intensity; Min-dB: minimum intensity; dB-range: range of intensity.
